# Natural Compounds for Counteracting GI and Liver Diseases

**DOI:** 10.3390/ijms27146475

**Published:** 2026-07-21

**Authors:** Raquel Abalo, Daniela Gabbia

**Affiliations:** 1 Department of Basic Health Sciences, University Rey Juan Carlos (URJC), 28922 Alcorcón, Spain; 2 High Performance Research Group in Physiopathology and Pharmacology of the Digestive System (NeuGut), University Rey Juan Carlos (URJC), 28922 Alcorcón, Spain; 3 Associated R+D+i Unit to the Institute of Medicinal Chemistry (IQM), Spanish National Research Council (CSIC), 28006 Madrid, Spain; 4 Working Group of Basic Sciences on Cannabinoids of the Spanish Pain Society, 28046 Madrid, Spain; 5 Working Group of Basic Sciences on Pain and Analgesia of the Spanish Pain Society, 28046 Madrid, Spain; 6 Department of Pharmaceutical and Pharmacological Sciences, University of Padova, 35131 Padova, Italy

Gastrointestinal (GI) and liver diseases remain among the most prevalent chronic disorders worldwide and represent a major burden for healthcare systems. Conditions such as gastroesophageal reflux disease (GERD), irritable bowel syndrome (IBS), chronic constipation, and metabolic dysfunction-associated steatotic liver disease (MASLD) are characterized by complex and multifactorial pathogenesis involving epithelial barrier dysfunction, chronic low-grade inflammation, oxidative stress, alterations in host–microbiota interactions, and metabolic dysregulation [1,2]. Recent evidence indicates that MASLD affects more than one-third of adults worldwide and has become the most common chronic liver disease globally, highlighting the urgent need for innovative preventive and therapeutic strategies [3]. Likewise, chronic gastrointestinal disorders, including disorders of gut–brain interaction (DGBIs), GERD, and functional bowel disorders, represent a major health challenge, affecting hundreds of millions of individuals worldwide, substantially impairing quality of life, increasing healthcare utilization, and generating a considerable socioeconomic burden [4,5,6]. These disorders are increasingly recognized as the consequence of complex interactions among epithelial barrier dysfunction, immune activation, gut microbiota alterations, visceral hypersensitivity, and dysregulated gut–brain communication rather than isolated organ-specific abnormalities [2,4,5,6].

In recent years, increasing attention has been devoted to natural compounds and naturally derived products as complementary or alternative therapeutic approaches. Advances in molecular biology, microbiome research, and systems medicine have provided new insights into the mechanisms through which these compounds exert biological effects. Rather than acting on a single molecular target, many natural products simultaneously influence inflammation, oxidative stress, epithelial integrity, microbial ecology, and cellular metabolism. Such pleiotropic activity is particularly relevant for chronic gastrointestinal and liver disorders, which are increasingly recognized as systemic conditions sustained by complex interactions among metabolic, immune, and microbial pathways.

Several common themes emerge across the contributions collected in this Special Issue: first, preservation of epithelial and mucosal barrier integrity appears as a central therapeutic target throughout the gastrointestinal tract; second, modulation of host–microbiota interactions and microbial metabolites represents a recurring mechanism linking nutrition, natural compounds, and disease outcomes; third, the biological activity of natural products is consistently characterized by multi-target actions involving inflammatory, oxidative, metabolic, and structural pathways. These observations align with current concepts describing the gut–liver axis as a dynamic network integrating microbial, metabolic, immune, and epithelial signals in both gastrointestinal and hepatic diseases. Disruption of structural and functional barriers along this axis is increasingly recognized as a key event contributing to disease progression, whereas restoration of barrier function has emerged as a promising therapeutic objective.

Among gastrointestinal disorders, preservation and restoration of mucosal integrity emerged as a central theme. In the study by Khalil et al., the authors investigated the protective properties of Poliprotect^®^, a substance-based medical device [2], using complementary in vitro and ex vivo experimental models [7]. This formulation, composed of a combination of polysaccharides, minerals, and flavonoids, protected epithelial cells from oxidative injury, preserved transepithelial electrical resistance under damaging conditions, and significantly reduced the loss of epithelial integrity induced by acidic and bile components in human esophageal biopsies. These findings reinforce the concept that impaired mucosal barrier function represents a key determinant of symptom generation and disease progression in GERD and related disorders. The importance of mucosal protection was further explored by Caterbi et al., who provided a comprehensive characterization of the bioadhesive, barrier-forming, buffering, and antioxidant properties of Poliprotect^®^, through a combination of cellular assays, electrophysiological analyses, systems biology approaches, and in vivo models [8]. Particularly noteworthy is the proposal of a non-pharmacological mechanism of action based on biophysical interactions with the mucosal surface rather than direct receptor-mediated effects.

The lower gastrointestinal tract disease was represented by two complementary contributions addressing functional bowel disorders and microbiota-related mechanisms. In their comprehensive review, Abalo et al. examined current evidence regarding herbal-based therapies for irritable bowel syndrome [9]. The authors critically evaluated both preclinical and clinical studies and identified peppermint oil and Iberogast among the interventions supported by the most robust clinical evidence, while also discussing emerging candidates such as curcumin, fennel oil, and cannabis-derived products. Importantly, the review highlights persistent limitations in the field, including methodological heterogeneity, small study populations, and insufficient long-term safety data. These observations are particularly relevant in light of the contemporary pathophysiology of IBS, which increasingly emphasizes the interplay among dietary factors, gut microbiota, immune activation, epithelial dysfunction, and gut–brain signaling. The progressive transition from symptom-based management toward mechanism-based and phenotype-oriented approaches may ultimately facilitate the development of personalized therapeutic strategies, including herbal-based interventions.

The study by Zhang et al. investigated the effects of five structurally distinct soluble dietary fibers in a murine model of constipation [10]. Although all fibers improved constipation-related parameters to some extent, marked differences emerged according to their monosaccharide composition and glycosidic linkages; these structural characteristics influenced microbial community composition, short-chain fatty acid production, gastrointestinal hormone profiles, and intestinal transit. The study therefore provides mechanistic evidence that the physiological effects of dietary fibers cannot be considered interchangeable and that specific structural features determine distinct biological outcomes. Consequently, the design of microbiota-targeted nutritional interventions may increasingly rely on the structural characteristics of specific fiber classes rather than on broad dietary fiber categories.

The liver contribution to this collection addresses one of the most rapidly growing challenges in hepatology, namely MASLD. Current models of disease pathogenesis recognize the central contribution of insulin resistance, adipose tissue dysfunction, chronic inflammation, oxidative stress, and gut-derived signals, all of which interact within the framework of the gut–liver axis. In this context, Besné-Eseverri et al. evaluated extracts obtained from different parts of *Opuntia stricta* var. *dillenii*, including fruit-derived by-products, in murine and human hepatocyte models of steatosis [11]. All tested extracts reduced intracellular triglyceride accumulation, with peel extracts showing particularly pronounced activity. The anti-steatotic effects were associated with inhibition of *de novo* lipogenesis and downregulation of the fatty acid transporter CD36. Beyond identifying a novel source of bioactive compounds with potential relevance for metabolic liver disease, this study also highlights the value of agricultural by-products as sustainable sources of health-promoting molecules.

In conclusion, the articles collected in this Special Issue illustrate the complexity of current research on natural compounds for gastrointestinal and liver diseases. Collectively, they support the concept that naturally derived products can influence disease-relevant pathways ranging from epithelial barrier maintenance and microbiota modulation to hepatic lipid metabolism and inflammatory regulation. Future investigations integrating omics technologies, systems biology, microbiome profiling, and well-designed clinical trials will be essential to identify responsive patient populations and translate these promising findings into precision medicine strategies for gastrointestinal and liver disorders.
